# hERG1/Kv11.1 activation stimulates transcription of p21^waf/cip^ in breast cancer cells *via* a calcineurin-dependent mechanism

**DOI:** 10.18632/oncotarget.3797

**Published:** 2015-04-12

**Authors:** Mathew Perez-Neut, Vidhya R. Rao, Saverio Gentile

**Affiliations:** ^1^ Department of Molecular Pharmacology and Therapeutics, Loyola University Chicago, Maywood, IL, USA

**Keywords:** p21waf/cip, hERG1/Kv11.1, breast cancer, calmodulin, Ca2+-dependent transcription

## Abstract

The function of Kv11.1 is emerging in breast cancer biology, as a growing body of evidence indicates that the hERG1/Kv11.1 potassium channel is aberrantly expressed in several cancer types including breast cancers.

The biological effects of Kv11.1 channel blockers and their associated side effects are very well known but the potential use of Kv11.1 activators as an anticancer strategy are still unexplored. In our previous work, we have established that stimulation of the Kv11.1 potassium channel activates a senescent-like program that is characterized by a significant increase in tumor suppressor protein levels, such as p21^waf/cip^ and p16^INK4A^. In this study we investigated the mechanism linking Kv11.1 stimulation to augmentation of p21^waf/cip^ protein level. We have demonstrated that the Kv11.1 channel activator NS1643 activates a calcineurin-dependent transcription of p21^waf/cip^ and that this event is fundamental for the inhibitory effect of NS1643 on cell proliferation. Our results reveal a novel mechanism by which stimulation of Kv11.1 channel leads to transcription of a potent tumor suppressor and suggest a potential therapeutic use for Kv11.1 channel activators.

## INTRODUCTION

The human ether a-go-go related gene 1 (hERG1) encodes for the voltage gated Kv11.1 potassium channel which is traditionally known to play a fundamental role in controlling membrane potential in neurons, myocytes [1] or in gland cells [2–4]. Interestingly, Kv11.1 has been found to be expressed in several cancer cell types including carcinomas of the breast in which Kv11.1 current activity appears to be essential for cell proliferation [5–8]. Although these data suggest that Kv11.1 has oncogenic properties, very little is known about the contribution of Kv11.1 activity to the mechanisms controlling cell cycle.

The *CDKN1A* gene encodes for the cyclin-dependent kinase inhibitor p21^waf/cip^ which plays a fundamental role in controlling cell-cycle progression [9]. It is generally accepted that p21^waf/cip^ transcription is activated by oncogenic stimuli to prevent dysregulation of the cell cycle process. By inhibiting the activity of cyclins, p21^waf/cip^ determines the arrest of the cell cycle progression at G1 and S-phase [10]. Changes of p21^waf/cip^ protein level can be controlled mostly at the transcriptional level and depend on cellular context. In quiescent cells, increased p21^waf/cip^ protein level is temporary as these cells are waiting for the proliferative stimuli that induces inhibition of p21^waf/cip^ synthesis [11]. In contrast, increased p21^waf/cip^ protein level that is associated with augmentation of p16^INK4A^ and beta-galactosidase, lead to a permanent arrest of the cell cycle as they become a part of the activation of a senescence program [12]. However, recent investigations have also revealed that activation of p21^waf/cip^ can be associated with tumorigenesis [13] or apoptosis [14].

Generally, p21^waf/cip^ transcription can occur via activation of tumor protein 53 (p53-dependent) and p53-independent mechanisms [15]. For example, hyperactive Ras or Raf determine p53-dependent transcription of p21^waf/cip^ via activation of the E2F1 transcription factor [16, 17]. Mutations in the p53 gene leading to the production of non-functional protein greatly increases the risk of developing breast cancer due to lack of p21^waf/cip^ function. In contrast, nuclear receptors such as androgen receptors can activate p21^waf/cip^ transcription independently of p53 by directly binding *CDKN1A* gene [18–20]. Interestingly, Ca^2+^ appears to cooperate with specific transcription factors such as SP1, AP2 or NFAT in transcribing p21^waf/cip^ independently of p53 [21].

Calcineurin (CN) is a ubiquitously expressed serine/threonine phosphatase that plays a fundamental role in many physiological or pathological states [22–24]. CN is activated by calmodulin upon increased intracellular Ca^2+^ and acts on numerous substrates including transcriptor factors such as the nuclear factor of activated T cell (NFAT) proteins. Therefore, CN is considered as a major player in the signaling pathways that couple changes in Ca^2+^ homeostasis and gene transcription. However, the role of CN in cancer biology is still debated as the effects of its activation in cells can favor tumor progression or suppression [25, 26].

In our previous works, we have demonstrated that application of hERG1/Kv11.1 potassium channel activator NS1643 to breast cancer cells caused an increase in Ca^2+^ entry [6] and increased p21^waf/cip^ protein level that resulted in a senescence-like phenotype while no effect was observed in non-transformed cells [6, 7]. However, we did not investigate the mechanism by which NS1643 augmented p21^waf/cip^ protein level. In this work we demonstrate that NS1643 stimulates p21^waf/cip^ transcription via activation of CN.

## RESULTS

### Stimulation of hERG1/Kv11.1 channel activity increases p21^waf/cip^ protein level in molecularly diverse breast cancer cells

In our previous work we have shown that chronic stimulation of hERG1/Kv11.1 potassium channel determined a strong increase of p21^waf/cip^ protein level in breast cancer cells that lack expression of estrogen receptor (ERneg). In the current study we have expanded our investigation on the effect of chronic stimulation of Kv11.1 on the tumor suppressor p21^waf/cip^ by testing the effect of NS1643 on molecularly different breast cancer cells representative of the following phenotype: Luminal A and p53-positive (MCF7), Luminal A and p53-negative (T47D), claudin-low breast cancer cells (MDA-MB-231) and HER2-overexpressing (SKBr3) breast cancer cells [27].

**Figure 1:**
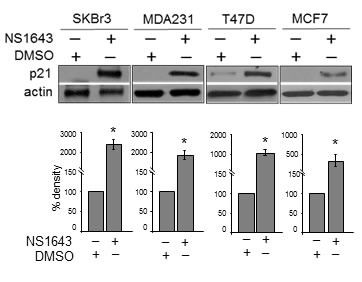
NS1643 increases p21^waf/cip^ protein level in breast cancer cells **A.** Representative western blot analyses of extracts (n=3; 32μg loading) from SKBr3, MDA-MB-231 (MDA231), T47D or MCF7 cells treated with DMSO (control) or NS1643 (50 μM) for 48 hr using p21 antibody. Anti-actin immunoblot was used as a loading control. Antibody were purchased from Cell Signaling. **B.** Bar graphs summarizing the observed effects of NS1643 exposure on p21 protein expression (*n* = 3; *p < 0.01). SKBr3 (HER2^+^, ER-, PR-, p53-null), MDA-MB-231 (HER2^+^, ER-, PR-, p53-null), MCF7 (HER2^+^, ER+, PR+, p53-WT), T47D (HER2^+^, ER+, PR+, p53-null) breast cancer cells were exposed to NS1643 (50μM) for 24 hr.

We found that application of NS1643 determined a significant increase of p21^waf/cip^ protein level that was similar in all breast cancer cells that we have examined (Figure 1). This suggests that the stimulation of Kv11.1 channel increases p21^waf/cip^ expression level independently from molecular heterogeneity of breast cancers.

### Suppression of p21^waf/cip^ function counteracts the inhibitory effects of NS1643 on cell proliferation

**Figure 2:**
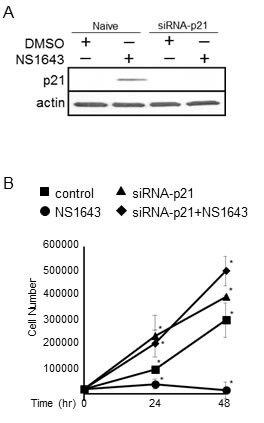
Suppression of p21^waf/cip^ counteracts the inhibitory effect of NS1643 on cell proliferation **A.** Immunoblot analysis showing efficacy of siRNA-mediated p21^waf/cip^ knockdown in SKBr3 cells. The blot shows the effect of NS1643 on p21^waf/cip^ in naïve cells (control) and in transfected cells. Actin immunoblots are utilized as loading controls (lower panels). **B.** Graph comparing the cell number over time of SKBr3 cells expressing siRNA targeting p21^waf/cip^ alone (siRNA-p21^waf/cip^; square), siRNA-p21^waf/cip^ and NS1643 (circle, 50 μM), NS1643 alone (diamond) or DMSO (control; triangle). (*n* = 6 each treatment; *p < 0.01).

To understand the contribution of p21^waf/cip^ in mediating the inhibitory effect of chronic stimulation of Kv11.1 channel on cancer cell growth we monitored the proliferation rate of cells in which p21^waf/cip^ function have been suppressed (Figure 2A) and treated with NS1643. Interestingly, the inhibitory effect of NS1643 on cell proliferation was completely repressed in cells expressing siRNA targeting p21^waf/cip^ compared to naïve cells (Figure 2B). These data suggest that p21^waf/cip^ plays a fundamental role in controlling the inhibitory effect of NS1643 on cancer cell growth.

### NS1643 induces p21 gene transcription

**Figure 3:**
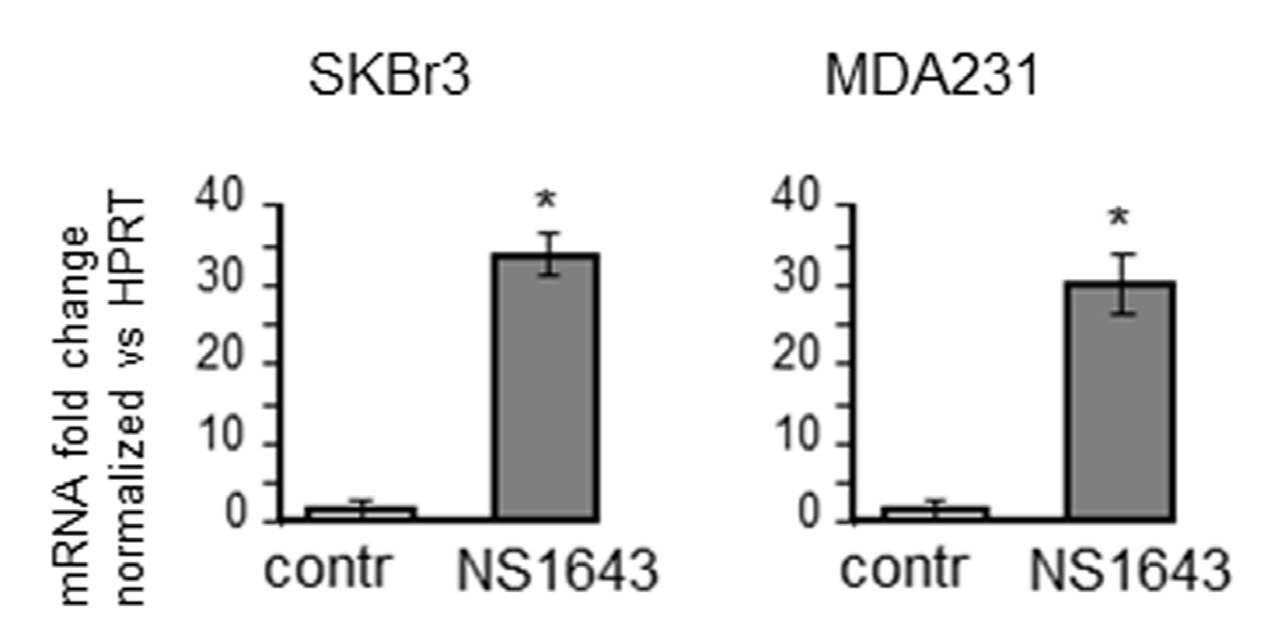
NS1643 increases p21 mRNA level Graph showing fold changes of p21 mRNA level in SKBr3 or MDA-MB-231 cells treated with or without NS1643 (50μM) for 24hr. *p* < 0.001 compared with control.

We first assessed whether the NS1643-dependent increased p21^waf/cip^ was a consequence of augmented protein synthesis. Therefore, we monitored mRNA level for p21^waf/cip^ in cells that were treated with or without Kv11.1 agonist (Figure 3). We found that application of NS1643 for 24hr determined a strong increase of mRNA level suggesting that activation of Kv11.1 channel stimulates p21^waf/cip^ gene induction.

**Figure 4:**
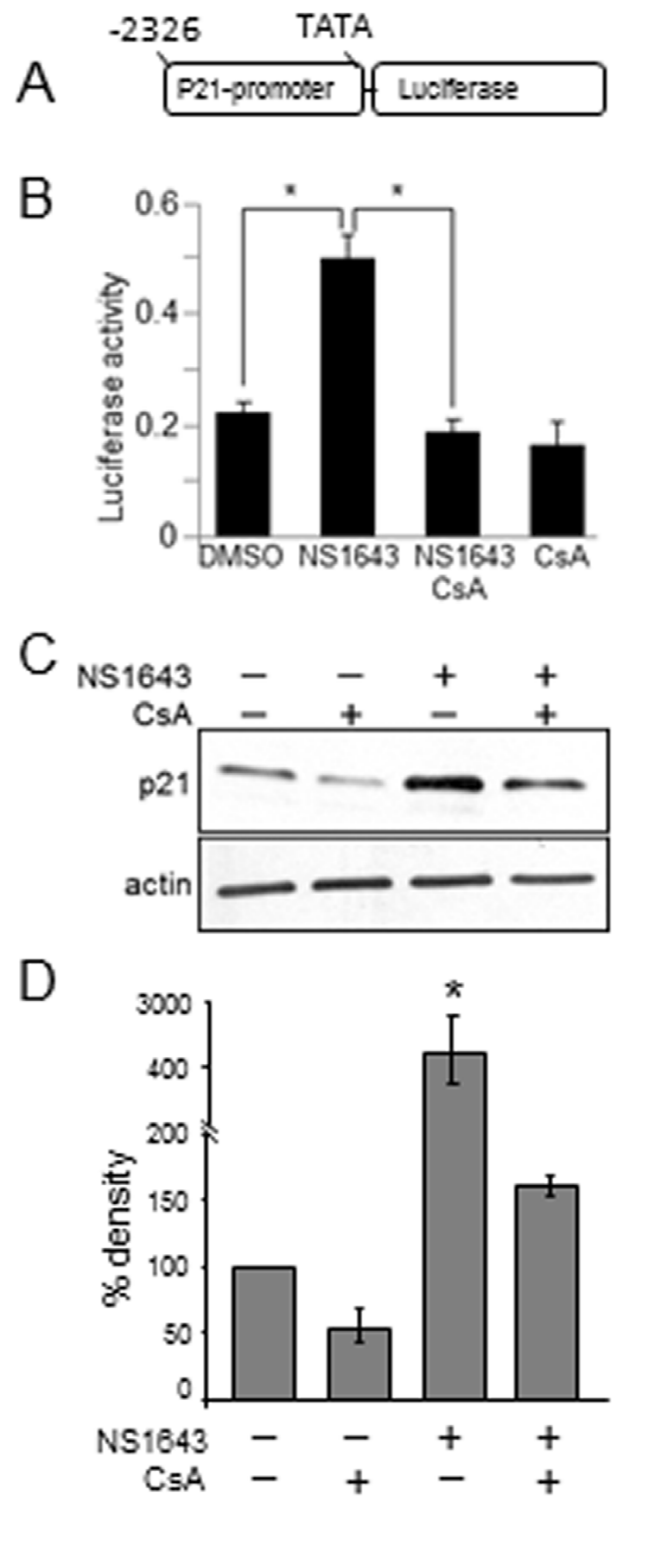
Cyclosporine-A inhibits NS1643-dependent stimulation of p21 induction **A.** Schematic illustration of the luciferase-based reporter construct that contains p21^waf/cip^-promoter region (Addgene plasmid #21723). **B.** Fold changes of p21-luciferase activity in SKBr3 expressing p21-luciferase reporter and treated with DMSO (control), NS1643, NS1643+Cyclosporine-A (CsA, 10 μM) or CsA alone. **C.** Western blot analyses of extracts from SKBr3 cells treated with CsA alone, NS1643 alone or CsA+NS1643 for 24hr. NS1643 for the indicated times, using p21 antibody. Antibody were purchased from Cell Signaling.

To further validate this hypothesis we transfected cells with a luciferase-based reporter construct that contains p21^waf/cip^-promoter region [28] and then we treated these cells with NS1643 (Figure 4A). We found that stimulation of Kv11.1 channel strongly induced the p21^waf/cip^ promoter as showed by increased luciferase activity.

### NS1643-dependent increase of p21 protein level is blocked by calcineurin inhibition

It is well understood that changes in cytosolic Ca^2+^ can activate the serine/threonine protein phosphatase calcineurin which in turn can activate biochemical pathways including nuclear transcription. Therefore, based on our previous discovery that NS1643 can increase cytosolic calcium [7], we monitored the effect of the calcineurin inhibitor cyclosporine A (CsA) on NS1643-dependent p21^waf/cip^ expression.

Strikingly, our luciferase assay revealed that CsA significantly blocked NS1643-dependent induction of p21^waf/cip^ gene (Figure 4A). Similarly, western blot analysis showed that application of CsA strongly impairs NS1643 to increase p21^waf/cip^ protein level Figure 4B). Altogether, these data suggest that stimulation of Kv11.1 potassium channel determine increased p21^waf/cip^ protein synthesis that is mediated by CN.

### NS1643 determines CN-dependent NFAT activation

To further test the hypothesis that the effect of NS1643 on p21^waf/cip^ protein level is mediated by CN-dependent gene transcription we monitored the activity of one of the best characterized CN-controlled gene regulators, NFAT [29, 30]. Consequently, to assess whether NFAT is activated by NS1643, we transfected cells with a luciferase-based reporter construct that contains NFAT response elements (NFAT-RE) [31] and we discovered that treating transfected cells with NS1643 strongly stimulates NFAT activation. Application of cobalt (Co^2+^), a generic surface membrane Ca^2+^ channel blocker, or the calcineurin blocker cyclosporine A (CsA) strongly inhibited the effect of NS1643 on NFAT activity suggesting that stimulation of the Kv11.1 channel activity leads to activation of NFAT-dependent transcription.

Furthermore, we assessed the ability of NS1643 in activating NFAT by monitoring nuclear translocation of endogenous NFAT. By using immunofluorescence/confocal microscopy we discovered that application of NS1643 determined an increased localization of the endogenous NFAT in the nucleus. To confirm this hypothesis, we performed western blot analysis of nuclear or cytoplasmic cell fractions from cells treated with NS1643. We found that application of NS1643 strongly increased protein level in the nuclear fraction compared to untreated cells.

These data indicate that stimulation of Kv11.1 channel promotes CN-dependent NFAT nuclear translocation and suggest that NFAT might contribute to the NS1643-dependent p21^waf/cip^ gene induction.

**Figure 5:**
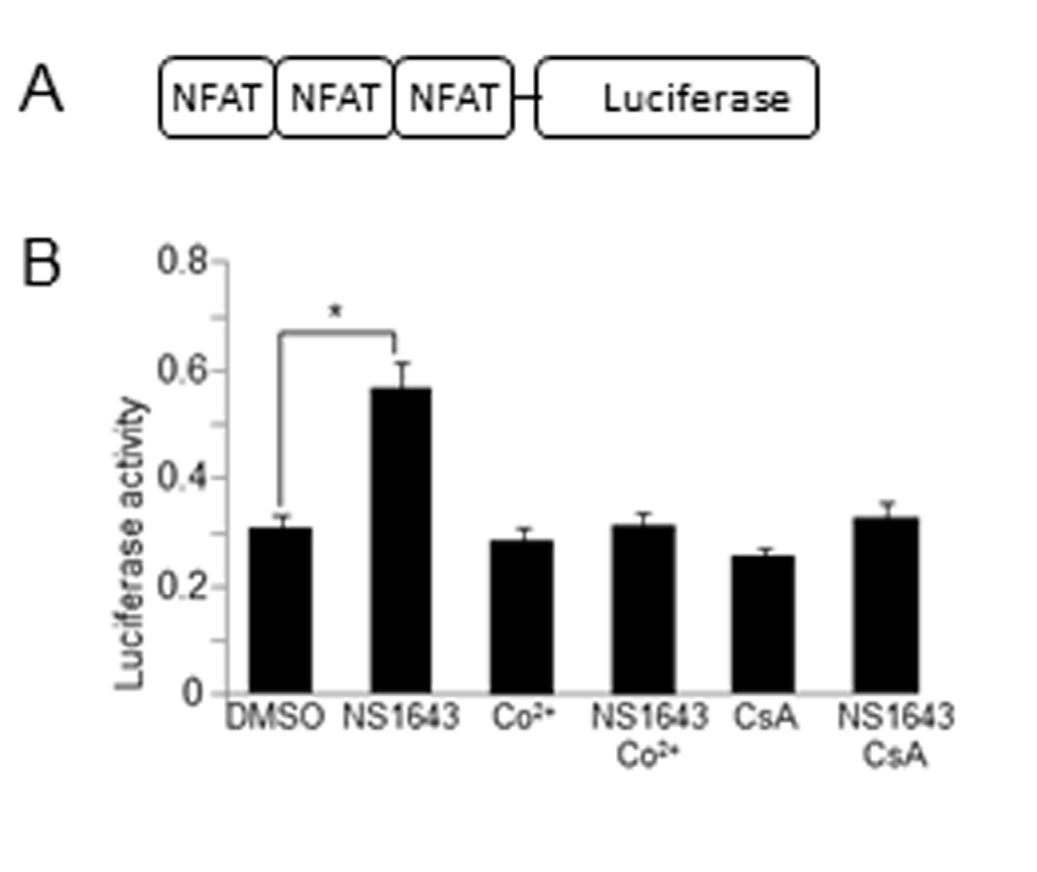
NS1643 stimulates NFAT-dependent gene induction **A.** Schematic illustration of the luciferase-based reporter construct that contains NFAT response elements (Addgene plasmid #10959) **B.** Fold changes of NFAT luciferase activity in SKBr3 expressing NFAT luciferase reporter and treated with DMSO (control), NS1643, Cobalt (Co+, 10 μM), NS1643+Co^2+^, Cyclosporine-A (CsA) or NS1643+CsA.

## DISCUSSION

Recent anticancer pharmacological approaches that have been developed into successful strategies have strongly reduced mortality related to breast cancers. For example, the discovery that anti-estrogens such as Tamoxifen can effectively inhibit the proliferative effects of estrogen (E2) on breast cancer cells dramatically improved breast cancer therapy. However, breast cancer is still the second leading cause of cancer death in women. Several reasons can account for the inefficacy of anti-breast cancer therapy. For example, a substantial fraction of breast cancers do not express estrogen receptors (ER, ER-negative) or can develop resistance to the therapeutic drug. This indicates that, molecular heterogeneity among breast cancers poses a severe limitation to therapy and underlines the urgent need for better pharmacologic approaches.

In our previous works, we discovered that use of a Kv11.1 channel activator (NS1643) determined a cell cycle arrest by activation of a senescence program, which coincided with a strong increase of the tumor suppressor p21^waf/cip^ and p16^INK4A^ protein level. In contrast, no significant consequences were observed in non-transformed cells following NS1643 application suggesting that, the effect of NS1643 was not due to the activity of the drug on unrelated/unreported targets. However, we did not explore the mechanism linking stimulation of the Kv11.1 potassium channel with expression of tumor suppressors.

In this study we have assessed that, the stimulating effects of NS1643 on p21^waf/cip^ protein level occurs in several types of breast cancer cell lines that are categorized by different molecular characteristics. For example, NS1643 application increased p21 protein level independently of the expression of ER or HER2. This suggests that, use of NS1643 as a potential anticancer molecule could be beneficial for treating breast cancers that are molecularly distinct.

The mechanism by which NS1643 increases p21 protein level appears to be related to a p53-independent activation of p21 gene transcription. In our experiments we tested the effect of NS1643 on p21 in several breast cancer cells lines including those in which mutations in the p53 gene rendered p53 non-functional (T47D; SKBr3; MDA-MB-231) or in cells in which was wild p53 was expressed (MCF7). We found that the effect of NS1643 was similar in all the cell lines suggesting that, the possible contribution of p53 in mediating the effect of NS1643 on p21 is minimal or irrelevant.

Our RT-PCR and luciferase assay investigations revealed that NS1643 application strongly increases p21^waf/cip^ transcription. Interestingly, this event appears to be controlled by Ca^2+^ entry as application of Co^2+^ (a generic surface Ca^2+^ channel blocker) completely inhibited the effect of NS1643 on p21^waf/cip^ transcription.

How can stimulation of a potassium channel drive Ca^2+^-dependent gene transcription?

We have previously shown that stimulation of Kv11.1 potassium channel results in build-up of an intracellular negative charge (hyperpolarization) resulting from the outflow of positive charged ions due to activation of the Kv11.1 potassium channel. Correspondingly, Hernandez et al. [32] found that inhibition of the Kv11.1 channel activity in osteoblastic osteosarcoma cells with the selective blocker E4031 determined membrane depolarization. Interestingly, although the effects of the activator and the blocker on the membrane potential were diametrically opposed, they both lead to increased cytosolic calcium and inhibition of cell proliferation. In the case of breast cancer cells NS1643-dependent hyperpolarization allows passive moving of Ca^2+^ ions from the extracellular solution into the cytoplasm. Interestingly, osteoblastic osteosarcoma cells express the L-type voltage-gated calcium channel (VGCC) [33]. Opening of this channel is dictated by depolarization. Therefore, E4031 can stimulate calcium entry via activation of the L-type calcium channel. Then, in osteoblastic osteosarcoma cells and breast cancer cells, Ca^2+^ activates a plethora of intracellular signaling, including membrane and/or nuclear biochemical cascades (e.g. NFAT) that can lead to a common outcome such as inhibition of cell proliferation.

**Figure 6:**
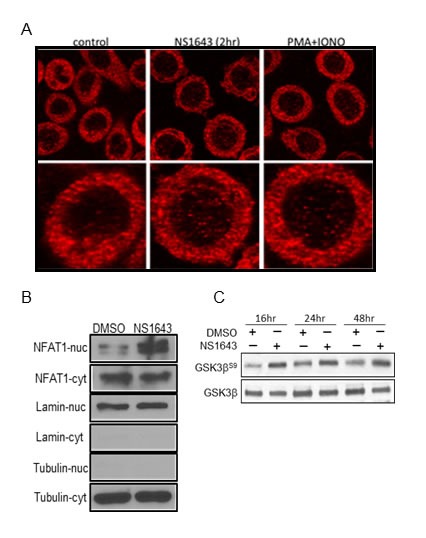
NS1643 promotes NFAT nuclear translocation **A.** NFAT (red) nuclear translocation in SKBR-3 cells with or without treatment with NS 1643 as observed my confocal microscopy. PMA (100 nM)/ionomycin (2 μM) treated cells served as positive control. **B.** Quantification of NFAT nuclear translocation by image J in control cells, cells treated with NS 1643 and cells treated with PMA/Ionomycin. Data expressed as Mean ± SEM (*N* = 37 cells). *Significant at *p* < 0.05 **C.** Nuclear and cytosol localization of NFAT in SKBr-3 cells treated with or without NS 1643 as observed by nucleus and cytosol fractionation and Western blot. The nucleus and cytosol extracts did not reveal any contamination as observed by the lack of tubulin in the nucleus and laminin in cytosol extracts. **D.** Western blot analyses of extracts from SKBr3 cells treated with DMSO (control) or NS1643 for the indicated times, using Phospho-GSK3β (Ser9) antibody (upper panel) or GSK3β (lower panel). Antibody were purchased from Cell Signaling.

Interestingly a study from Hegle et al. showed that gating of Kv10.1 potassium channel, which belongs to the same superfamily of the Kv11.1 channel, can stimulate proliferation in cancer cells by a mechanism that is independent from K^+^ flux through the channel and/or changes in intracellular calcium [34]. This suggests that members of the EAG superfamily of potassium channels can influence cell proliferation independently form their ability to control membrane potential. However, previous investigations conducted by the Mitcheson's Lab have clearly indicated that, in contrast to EAG channel, the oncogenicity exhibited by the hERG potassium channel appears to be strictly dependent by its ion conduction. In addition, in our experiments, changes of intracellular calcium concentration are crucial for the effects of NS1643 on cell proliferation. We think that altogether, our data suggests that conduction properties of Kv11.1 are fundamental for determining the inhibitory effects upon Kv11.1 chronic activation.

**Figure 7:**
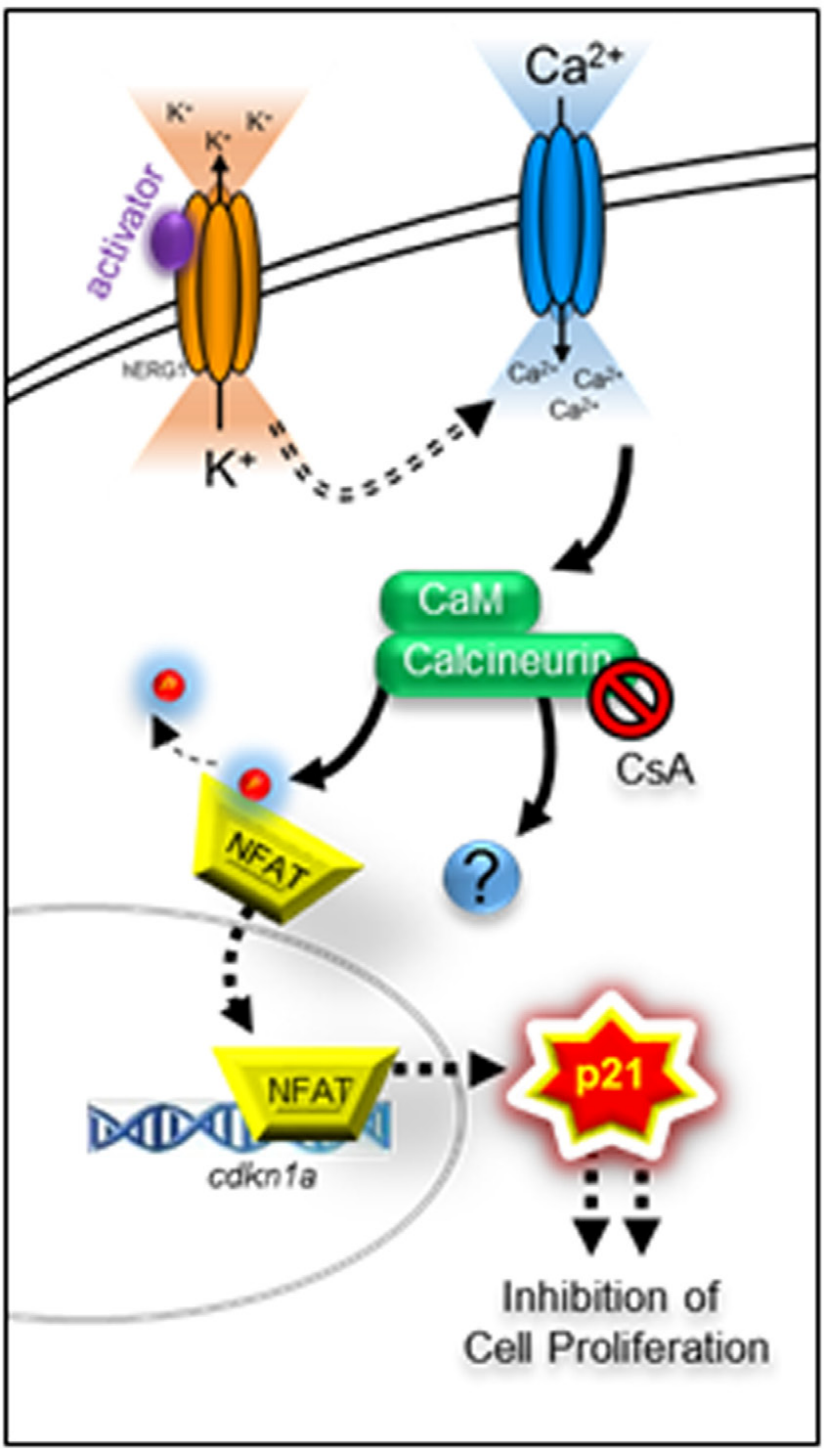
Schematic representation of hERG1/Kv11.1 activator-dependent stimulation of p21^waf/cip^ Stimulation of hERG1/Kv11.1 potassium channel with NS1643 increases passive Ca^2+^ entry due to hyperpolarization of the cell. Augmented intracellular Ca^2+^ stimulates CN which induces *CDKN1* gene transcription via activation of transcription factor(s) i.e. members of the NFAT family.

The protein phosphatase CN plays a multifunctional role in cell biology by linking the interaction between Ca^2+^/calmodulin to activation of transcription factors. These events have been originally studied in neurons or immune cells but the discovery that, the immunosuppressant cyclosporine A (CsA) targets CN, provided pharmacological reagents that allowed to demonstrate that, CN plays a major role in “sensing” calcium homeostasis in all eukaryotic cells.

Interestingly, results from our experiments revealed that, the effect of NS1643 on p21^waf/cip^ gene induction is completely inhibited by application of CsA suggesting that, CN can play an important role in mediating the effects of Kv11.1 activation via p21^waf/cip^ gene induction. Nevertheless, we cannot rule out at this time that CN could also modulate other mechanisms that for example control mRNA stability and more defined experiments are needed.

P53-independent regulation of p21^waf/cip^ expression involves several Ca^2+^-activated transcription factors including four of the five members of the NFAT family (NFAT1-4). NFAT1-4 exist inactive in the cytoplasm as phosphorylated proteins. Ca^2+^ entry stimulates calcineurin that dephosphorylates NFATs and allow its translocation into the nucleus. Then, NFATs can induce transcription of several genes either by acting as homo/hetero dimers, alone or in collaboration with other transcriptor factors such as AP1, SP3. Termination of NFAT signaling can occur by decreasing concentration of intracellular Ca^2+^ or/and phosphorylation by nuclear kinases such as GSK3β resulting in subsequent NFAT translocation to the cytoplasm and/or degradation. Interestingly, our experiments revealed that NS1643 can strongly activate NFAT as revealed by luciferase assay, detection of nuclear translocation of the endogenous NFAT protein and inhibition of the NFAT negative regulator, GSK3β. These data suggest that, NFAT might contribute to the effect of NS1643 on p21^waf/cip^ in breast cancer cells. However, at this time we don't know which members of the NFAT family can regulate p21 transcription or if they cooperate with other Ca^2+^ activated transcriptor factors.

Several scientific reports have revealed that in human, cellular senescence is mostly driven by expression of 16^INK4A^ [35–37]. However, we do not know whether the role of 16^INK4A^ is also predominant in NS1643-activated cellular senescence.

Our experiments have revealed that, proliferative cells (untreated cells) express very low levels of p21^waf/cip^ or p16^INK4A^ (undetectable by western blot analysis). Nevertheless, cells in which p21^waf/cip^ function has been genetically suppressed, present an increased ability to proliferate compared to naïve cells. In our view this suggests that, even if expressed at a low level, p21^waf/cip^ can strongly influence proliferation.

In addition, our experiments have also revealed that, the inhibitory effect of NS1643 on cell proliferation was completely removed by suppressing p21^waf/cip^ function, suggesting that even though NS1643 treatment leads to upregulation of both p21^waf/cip^ and 16^INK4A^, the role of p21^waf/cip^ appears to predominate. However, we cannot rule out that 16^INK4A^ can still play a major role in NS1643-driven senescence by acting as a downstream effector of p21^waf/cip^ and better dedicated experiments need to be designed.

In conclusion, we think that, we have discovered a novel mechanism through which stimulation of hERG1 potassium channel leads to inhibition of breast cancer cell proliferation via activation of NFAT1. In addition, our results offers a compelling opportunity to study Ca^2+^-dependent mechanism in cancer biology which can provide basis for anticancer therapeutic strategies [38].

## MATERIALS AND METHODS

### Cell culture

SKBr3 were cultured in RPMI-1640 (Thermo Scientific). MDA-MB-231, T47D and MCF7 were cultured in DMEM (Corning). All media contained 10% FBS (Serum Source International) and 100 μl/ml penicillin/streptomycin (Thermo Scientific) with the exception of T47D that also contained 1x NEAA (Thermo Scientific). All cells were grown at 37°C in 5% CO_2._

### Western blot

SKBr3 cells were harvested for western blot analysis by trypsinization with 0.25 % trypsin-EDTA (Thermo Scientific). Cells were washed with 1x PBS (Thermo Scientific) and resuspended in cold radioimmunoprecipitation assay buffer (RIPA) 50 mM Tris HCl (pH 8.0), 150 mM NaCl, 1% Tergitol (Sigma-Aldrich, St. Louis, MO, USA), 0.5% Na-deoxycholate, 0.1% SDS, 1 mM phenylmethylsulfonyl fluoride, 1 mM NaF, 1 mM Na3VO4, and 1 × Halt Protease Inhibitor Cocktail (Thermo Scientific). Protein concentration was determined by BCA assay (Thermo Scientific). 4x Laemmli buffer containing 10% 2-mercaptoethanol was added to lysates and boiled at 95 °C for 5 minutes. Lysates were loaded on 8% SDS-PAGE gels and transferred onto nitrocellulose membranes (Bio-Rad).

### Nuclear/cytosol fractionation

Nuclear and cytosol extracts from SKBR3 cells following various treatments were prepared as described below. Cells were washed with 1X PBS and cell pellets were lysed with a hypotonic buffer (10 mmol/L HEPES buffer pH 7.9, 1.5 mmol/L MgCl2, 10 mmol/L KCl, 10% glycerol, 0.5 mmol/L phenylmethylsulfonyl fluoride (PMSF), and 1 mmol/L dithiothreitol (DTT)). The lysates were kept on ice for 15 min, 5μl of 10% NP 40 was added to each lysate, vortexed briefly followed by centrifugation at 13200 rpm for 10min to pellet the nuclear fractions. The supernatant was collected as the cytosol extract. The nuclear pellet were lysed with buffer solution containing 20 mmol/L HEPES buffer pH 7.9, 1.5 mmol/L MgCl2, 400 mmol/L NaCl, 0.2 mmol/L EDTA, 20% glycerol, 0.5 mmol/L PMSF, and 1 mmol/L DTT. The nuclear lysates were clarified by centrifugation at 13200 rpm for 10 min at 4°C.

### Immunofluorescence microscopy

SKBR3 cells were grown on poly D-Lysine coated coverslips and treated with vehicle alone, 50 μM NS1643 or 80nM PMA and 2μM Ionomycin in RPMI 1640 media. Following treatment, the cells were washed with 1X PBS, fixed with 4% paraformaldehyde for 30 min and permeabilized with 0.3% Triton X-100 for 15 min at room temperature. Nonspecific binding was blocked with 5% bovine serum albumin (BSA) for 1h at room temperature and then incubated with 1:100 primary rabbit monoclonal anti-NFAT-1 antibody (Cell signaling Technology, Danvers, MA) diluted in 1% BSA at 4°C, overnight, while cells incubated with 1% BSA alone, served as negative controls. Following incubation with the primary antibody, the cells were washed with 1X PBS and incubated with Alexa fluor 594 conjugated anti-rabbit secondary antibody (Life technoloiges, Carlsbad, CA). Following washes, cells were incubated with 50ng/mL DAPI to stain the nuclei. The coverslips were mounted on VECTASHIELD reagent (Vector Laboratories, Burlingame, CA) and fluorescent images were taken using confocal microscopy (Carl Zeiss Meditec, Inc., Thornwood, NY). Nuclear translocation of NFAT-1 across groups was analyzed and quantified using the analyze particle tool from Image J.

### RNA isolation, RT-PCR and quantitative PCR

Total RNA was isolated using TRIzol reagent (Invitrogen, Carlsbad, CA, USA) and cDNA was synthesized using iScript (Biorad, Life Science Research, Hercules, CA, USA). For p21 amplification, primers p21-F (5′-TGA GCC GCG ACT GTG ATG-3′) and p21-R (5′-GTC TCG GTG ACA AAG TCG AAG TT-3′) were used.

For HSRP amplification, primers HSRP-F (5′-AGC CTC AAG ATC ATC AGC AAT G-3′) and GAPDH-R (5′-ATG GAC TGT GGT CAT GAG TCC TT-3′) were used.

To determine p21 mRNA levels and stability, quantitative RT-PCR was performed on a Mastercycler^®^ ep Realpex (Eppendorf, Hauppauye, NY, USA) using a total volume of 20 μl containing 200 nM primers and 1X Absolute^™^ Blue QPCR SYBR^®^ Green Mix (ABgene, Thermo Fisher Scientific, Rockford, IL, USA).

### Luciferase assay

Dual-luciferase reporter assay was done according to the manufacturer's instructions (Promega, Madison, WI, USA). Briefly, cells (4 × 10^4^) were plated on a 24-well plate, for 24 hr.ells in triplicate wells were co-transfected with 5 μg plasmid DNA encoding firefly luciferase driven by the NFAT or p21 promoter and 1μg of DNA encoding a constitutively expressed Renilla luciferase gene.

The p21-promoter luciferase reporter luciferase plasmid was generated by Toren Finkel group. This construct was a gift from Toren Finkel (Addgene plasmid # 10959)

The luciferase reporter gene for the studies of NFAT activity contains three tandem repeats of a 30-bp fragment (GGAGG AAAAA CTGTT TCATA CAGAA GGCGT) of the IL-2. This construct was a gift from Wafik El-Deiry (Addgene plasmid # 21723).

The firefly luciferase plasmid contains a luciferase reporter gene driven by a tandem repeat of NFAT binding elements and served as reporter of NFAT activation. Renilla luciferase serves as the control reporter to normalize the activity of the experimental reporter based on transfection efficiency and cell viability. Two days after transfection, the cells were exposed to drugs as indicated. Cells were harvested, and lysates were tested for luciferase activity using a dual-luciferase assay system. Y axis indicates fold change of relative luminescence which denotes the ratio of firefly luciferase activity (NFAT-dependent) to Renilla luciferase (control) activity to standardize for transfection efficiency.

## References

[1] Vandenberg JI, Perry MD, Perrin MJ, Mann SA, Ke Y, Hill AP (2012). hERG K(+) channels: structure, function, and clinical significance. Physiol Rev.

[2] Gentile S, Martin N, Scappini E, Williams J, Erxleben C, Armstrong DL (2008). The human ERG1 channel polymorphism, K897T, creates a phosphorylation site that inhibits channel activity. Proc Natl Acad Sci U S A.

[3] Gentile S, Darden T, Erxleben C, Romeo C, Russo A, Martin N, Rossie S, Armstrong DL (2006). Rac GTPase signaling through the PP5 protein phosphatase. Proc Natl Acad Sci U S A.

[4] Binggeli R, Weinstein RC (1986). Membrane potentials and sodium channels: hypotheses for growth regulation and cancer formation based on changes in sodium channels and gap junctions. J Theor Biol.

[5] Pier DM, Shehatou GS, Giblett S, Pullar CE, Trezise DJ, Pritchard CA, Challiss RA, Mitcheson JS (2014). Long-term channel block is required to inhibit cellular transformation by human ether-a-go-go-related gene (hERG1) potassium channels. Mol Pharmacol.

[6] Perez-Neut M, Shum A, Cuevas BD, Miller R, Gentile S (2014). Stimulation of hERG1 channel activity promotes a calcium-dependent degradation of cyclin E2, but not cyclin E1, in breast cancer cells. Oncotarget.

[7] Lansu K, Gentile S (2013). Potassium channel activation inhibits proliferation of breast cancer cells by activating a senescence program. Cell Death Dis.

[8] Jehle J, Schweizer PA, Katus HA, Thomas D (2011). Novel roles for hERG K(+) channels in cell proliferation and apoptosis. Cell Death Dis.

[9] Gartel AL, Serfas MS, Tyner AL (1996). p21--negative regulator of the cell cycle. Proc Soc Exp Biol Med.

[10] Brugarolas J, Moberg K, Boyd SD, Taya Y, Jacks T, Lees JA (1999). Inhibition of cyclin-dependent kinase 2 by p21 is necessary for retinoblastoma protein-mediated G1 arrest after gamma-irradiation. Proc Natl Acad Sci U S A.

[11] Coller HA, Sang L, Roberts JM (2006). A new description of cellular quiescence. PLoS Biol.

[12] Munoz-Espin D, Serrano M (2014). Cellular senescence: from physiology to pathology. Nat Rev Mol Cell Biol.

[13] Shah MA, Kortmansky J, Motwani M, Drobnjak M, Gonen M, Yi S, Weyerbacher A, Cordon-Cardo C, Lefkowitz R, Brenner B, O'Reilly E, Saltz L, Tong W, Kelsen DP, Schwartz GK (2005). A phase I clinical trial of the sequential combination of irinotecan followed by flavopiridol. Clin Cancer Res.

[14] Gartel AL, Tyner AL (2002). The role of the cyclin-dependent kinase inhibitor p21 in apoptosis. Mol Cancer Ther.

[15] Abbas T, Dutta A (2009). p21 in cancer: intricate networks and multiple activities. Nat Rev Cancer.

[16] Jancik S, Drabek J, Radzioch D, Hajduch M (2010). Clinical relevance of KRAS in human cancers. J Biomed Biotechnol.

[17] Gartel AL, Najmabadi F, Goufman E, Tyner AL (2000). A role for E2F1 in Ras activation of p21(WAF1/CIP1) transcription. Oncogene.

[18] Gartel AL, Tyner AL (1999). Transcriptional regulation of the p21((WAF1/CIP1)) gene. Exp Cell Res.

[19] Macleod KF, Sherry N, Hannon G, Beach D, Tokino T, Kinzler K, Vogelstein B, Jacks T (1995). p53-dependent and independent expression of p21 during cell growth, differentiation, and DNA damage. Genes Dev.

[20] Saramaki A, Banwell CM, Campbell MJ, Carlberg C (2006). Regulation of the human p21(waf1/cip1) gene promoter via multiple binding sites for p53 and the vitamin D3 receptor. Nucleic Acids Res.

[21] Santini MP, Talora C, Seki T, Bolgan L, Dotto GP (2001). Cross talk among calcineurin, Sp1/Sp3, and NFAT in control of p21(WAF1/CIP1) expression in keratinocyte differentiation. Proc Natl Acad Sci U S A.

[22] Rusnak F, Mertz P (2000). Calcineurin: form and function. Physiol Rev.

[23] Aramburu J, Heitman J, Crabtree GR (2004). Calcineurin: a central controller of signalling in eukaryotes. EMBO Rep.

[24] Erxleben C, Liao Y, Gentile S, Chin D, Gomez-Alegria C, Mori Y, Birnbaumer L, Armstrong DL (2006). Cyclosporin and Timothy syndrome increase mode 2 gating of CaV1.2 calcium channels through aberrant phosphorylation of S6 helices. Proc Natl Acad Sci U S A.

[25] Dotto GP (2009). Calcineurin signaling as a negative determinant of keratinocyte cancer stem cell potential and carcinogenesis. Cancer Res.

[26] Wu X, Nguyen BC, Dziunycz P, Chang S, Brooks Y, Lefort K, Hofbauer GF, Dotto GP (2010). Opposing roles for calcineurin and ATF3 in squamous skin cancer. Nature.

[27] Holliday DL, Speirs V (2011). Choosing the right cell line for breast cancer research. Breast Cancer Res.

[28] Zeng YX, Somasundaram K, el-Deiry WS (1997). AP2 inhibits cancer cell growth and activates p21WAF1/CIP1 expression. Nat Genet.

[29] Crabtree GR, Olson EN (2002). NFAT signaling: choreographing the social lives of cells. Cell.

[30] Mancini M, Toker A (2009). NFAT proteins: emerging roles in cancer progression. Nat Rev Cancer.

[31] Ichida M, Finkel T (2001). Ras regulates NFAT3 activity in cardiac myocytes. J Biol Chem.

[32] Hernandez L, Park KH, Cai SQ, Qin L, Partridge N, Sesti F (2007). The antiproliferative role of ERG K+ channels in rat osteoblastic cells. Cell Biochem Biophys.

[33] Grygorczyk C, Grygorczyk R, Ferrier J (1989). Osteoblastic cells have L-type calcium channels. Bone Miner.

[34] Hegle AP, Marble DD, Wilson GF (2006). A voltage-driven switch for ion-independent signaling by ether-a-go-go K+ channels. Proc Natl Acad Sci U S A.

[35] Krishnamurthy J, Ramsey MR, Ligon KL, Torrice C, Koh A, Bonner-Weir S, Sharpless NE (2006). p16INK4a induces an age-dependent decline in islet regenerative potential. Nature.

[36] Rayess H, Wang MB, Srivatsan ES (2012). Cellular senescence and tumor suppressor gene p16. Int J Cancer.

[37] Hara E, Smith R, Parry D, Tahara H, Stone S, Peters G (1996). Regulation of p16CDKN2 expression and its implications for cell immortalization and senescence. Mol Cell Biol.

[38] Huang X, Jan LY (2014). Targeting potassium channels in cancer. J Cell Biol.

